# Vital Signs: *Listeria* Illnesses, Deaths, and Outbreaks — United States, 2009–2011

**Published:** 2013-06-07

**Authors:** Benjamin J. Silk, Barbara E. Mahon, Patricia M. Griffin, L. Hannah Gould, Robert V. Tauxe, Stacy M. Crim, Kelly A. Jackson, Peter Gerner-Smidt, Karen M. Herman, Olga L. Henao

**Affiliations:** Div of Foodborne, Waterborne, and Environmental Diseases, National Center for Emerging and Zoonotic Infectious Diseases, CDC

## Abstract

**Background:**

Older adults, pregnant women, and persons with immunocompromising conditions are at higher risk than others for invasive *Listeria monocytogenes* infection (listeriosis), a rare and preventable foodborne illness that can cause bacteremia, meningitis, fetal loss, and death.

**Methods:**

This report summarizes data on 2009–2011 listeriosis cases and outbreaks reported to U.S. surveillance systems. The *Listeria* Initiative and PulseNet conduct nationwide surveillance to rapidly detect and respond to outbreaks, the Foodborne Diseases Active Surveillance Network (FoodNet) conducts active, sentinel population–based surveillance to track incidence trends, and the Foodborne Disease Outbreak Surveillance System (FDOSS) receives reports of investigated outbreaks to track foods and settings associated with outbreaks.

**Results:**

Nationwide, 1,651 cases of listeriosis occurring during 2009–2011 were reported. The case-fatality rate was 21%. Most cases occurred among adults aged ≥65 years (950 [58%]), and 14% (227) were pregnancy-associated. At least 74% of nonpregnant patients aged <65 years had an immunocompromising condition, most commonly immunosuppressive therapy or malignancy. The average annual incidence was 0.29 cases per 100,000 population. Compared with the overall population, incidence was markedly higher among adults aged ≥65 years (1.3; relative rate [RR]: 4.4) and pregnant women (3.0; RR: 10.1). Twelve reported outbreaks affected 224 patients in 38 states. Five outbreak investigations implicated soft cheeses made from pasteurized milk that were likely contaminated during cheese-making (four implicated Mexican-style cheese, and one implicated two other types of cheese). Two outbreaks were linked to raw produce.

**Conclusions:**

Almost all listeriosis occurs in persons in higher-risk groups. Soft cheeses were prominent vehicles, but other foods also caused recent outbreaks. Prevention targeting higher-risk groups and control of *Listeria monocytogenes* contamination in foods implicated by outbreak investigations will have the greatest impact on reducing the burden of listeriosis.

**Implications for Public Health Practice:**

Careful attention to food safety is especially important to protect vulnerable populations. Surveillance for foodborne infections like listeriosis identifies food safety gaps that can be addressed by industry, regulatory authorities, food preparers, and consumers.

## Introduction

*Listeria monocytogenes* infection (listeriosis), recognized as a foodborne illness in the 1980s (1), leads to invasive disease during vulnerable stages of life (2). Older adults and persons with immunocompromising conditions are at higher risk for *Listeria* bacteremia and meningitis (3), which can be fatal. Listeriosis usually is a mild illness in pregnant women, but it can cause severe outcomes for the fetus or newborn infant, including fetal loss, preterm labor, and neonatal sepsis, meningitis, and death. Listeriosis is rare (3). However, hospitalization is much more common than with other foodborne infections (4), and listeriosis is the third leading cause of death among major pathogens transmitted commonly by food (5). Listeriosis incidence decreased by 24% from 1996 through 2001 but has not changed significantly since then (3,4). Although most cases are sporadic (i.e., not outbreak-related) (6), outbreaks occur regularly (7). In 2011, contaminated cantaloupe from a single farm caused the deadliest U.S. foodborne disease outbreak in nearly 90 years (8). Public health officials rapidly implicated whole cantaloupe, and their actions prevented additional cases and deaths. Outbreak investigations also can reveal unrecognized food sources and food safety gaps that can be closed by regulatory and industry intervention.

This report provides an overview of recent surveillance data on listeriosis, highlighting actions needed to protect vulnerable populations.

## Methods

The objectives of this report are to 1) summarize demographic and clinical characteristics of patients with listeriosis, 2) estimate incidence overall and in demographic subgroups, and 3) describe foods associated with outbreaks. Data from three surveillance systems for the period 2009–2011 were analyzed to provide this comprehensive picture. A case of invasive listeriosis was defined as isolation of *L. monocytogenes* from a normally sterile site (e.g., blood or cerebrospinal fluid) or from products of conception. When *L. monocytogenes* was isolated from multiple sites, a single site is reported (priority order: cerebrospinal fluid, blood, other normally sterile site, products of conception). A case was considered pregnancy-associated when it occurred in a pregnant woman, a fetus, or an infant ≤31 days old; mother-infant pairs were counted as a single case. The case-fatality rate (CFR) was calculated as the percentage of cases with a fatality. Fetal losses were tallied separately from deaths but were included in CFR calculations. Live-born infants were assumed to have survived unless reported to have died.

The primary data source for the first objective was the *Listeria* Initiative,* a CDC-led enhanced nationwide surveillance system that collects demographic, clinical, and food exposure data for persons with laboratory-confirmed listeriosis. Patients are interviewed as they are reported, using a standard questionnaire. Isolates of *L. monocytogenes* from patients are subtyped in PulseNet,† the national molecular subtyping network. The *Listeria* Initiative facilitates investigation of possible outbreaks identified by PulseNet. *Listeria* Initiative participation has steadily improved since national implementation in 2005; 47 states reported at least one case in 2011.

Also for the first objective, the Foodborne Diseases Active Surveillance Network (FoodNet)§ contributed data on underlying conditions. FoodNet is a collaborative program among CDC, 10 state health departments, the U.S. Department of Agriculture’s Food Safety and Inspection Service (USDA-FSIS), and the Food and Drug Administration (FDA). FoodNet conducts active, population-based surveillance for laboratory-confirmed infections with *L. monocytogenes* and eight other pathogens among residents of 10 sites covering approximately 15% of the U.S. population (48 million persons in 2011). FoodNet does not routinely track underlying medical conditions; they can be reported voluntarily, but reporting is incomplete.

For the second objective, incidence rates were calculated by dividing FoodNet data on the number of laboratory-confirmed infections by U.S. Census estimates of the population of the surveillance area, both for the whole population and for subgroups. FoodNet and *Listeria* Initiative data were linked to improve completeness of information on ethnicity and pregnancy.

For the third objective, data from the Foodborne Disease Outbreak Surveillance System (FDOSS)¶ were used. State, local, and territorial health departments submit reports of investigated foodborne disease outbreaks to CDC. For each outbreak, FDOSS records the etiology, state(s), size (i.e., number of illnesses), setting, and food vehicle, among other data. A listeriosis outbreak was defined as ≥2 cases linked to a common source. Outbreaks were considered multistate if exposure to the implicated food occurred in more than one state.

## Results

Nationwide, 1,651 invasive listeriosis cases were reported to the *Listeria* Initiative from 2009 through 2011; 292 deaths or fetal losses were reported (CFR: 21%). Most (58%) cases were in adults aged ≥65 years, and 14% were pregnancy-associated (Table 1). The median age of patients with listeriosis that was not pregnancy-associated was 72 years (interquartile range [IQR]: 61–81 years). Among pregnancy-associated cases with ethnicity data available, 43% (85 of 198) of mothers were Hispanic. Preterm labor was reported in 64% of pregnancy-associated cases. Among nonpregnant patients aged <65 years reported to FoodNet, an underlying medical condition was recorded for 74% (96 of 130); immunosuppressive therapy (i.e., steroids, chemotherapy, or radiation) was most commonly reported (32 cases), followed by malignancy (24), diabetes mellitus (11), cirrhosis or liver disease (seven), renal failure or nephrotic syndrome (seven), alcoholism (six), and human immunodeficiency virus/acquired immunodeficiency syndrome (six).

The average annual incidence was 0.29 cases per 100,000 population in FoodNet. In adults aged ≥65 years, the incidence was 1.3 cases per 100,000 population. The highest rates were among pregnant women (3.0 per 100,000), especially Hispanics (7.0 per 100,000). Compared with the population as a whole, rates were four times higher for adults aged ≥65 years (RR: 4.4), 10 times higher for pregnant women (RR: 10.1), and 24 times higher for pregnant Hispanic women (RR: 24.0).

Twelve outbreaks, five of them multistate, and 224 outbreak-associated cases (14% of cases reported to the *Listeria* Initiative) were reported among residents of 38 states (Table 2). The median size was seven cases (range: two to 147 cases). In seven (58%), the implicated food was consumed primarily in private homes. Two were linked to hospital food services, one to a restaurant, and one to wedding banquets. Ten (83%) investigations implicated a food vehicle. Cheese was implicated in six outbreaks (50% of outbreaks) with 51 cases (23% of outbreak-associated cases). Soft cheeses labeled as made from pasteurized milk were implicated in five outbreaks: four implicated Mexican-style cheese and one implicated both chive cheese and ackawi cheese (a white brine cheese). An aged, blue-vein cheese made from unpasteurized milk was implicated in the sixth outbreak. Two raw produce items, pre-cut celery (an ingredient in chicken salad) and whole cantaloupe, were implicated as listeriosis outbreak vehicles.

## Conclusions and Comment

This report details the epidemiology of invasive listeriosis, which often leads to bacteremia, meningitis, hospitalization, fetal loss, and death, and calls for actions that could protect the most vulnerable populations. Older adults and pregnant women, particularly pregnant Hispanic women, are at much higher risk than the population at large, as are persons with weakened immunity (2). Preventing infections in these populations can have substantial impact in averting these outcomes. Older adults and persons with weakened immunity, as well as infants and young children, are also prone to many other foodborne illnesses, including campylobacteriosis, salmonellosis, and Shiga toxin–producing *E. coli* infections (4). Accounting for underdiagnosis and underreporting, an estimated 1,662 cases of listeriosis occur each year (5). No progress in reducing the overall incidence of listeriosis has occurred in over a decade (3,4); renewed prevention efforts are needed from farm to table.

Foods associated with listeriosis outbreaks in this report,** soft cheese and raw produce items in particular, highlight opportunities for food safety improvements. *Listeria* is widespread in many environments, and reducing contamination of soft cheese and raw produce with *Listeria* and other pathogens will require implementation of proven measures as well as development of new ones. The Food Safety Modernization Act (FSMA) of 2011†† gives FDA additional authority to regulate food facilities, establish standards for safe produce, recall contaminated foods, and oversee imported foods. FDA has proposed new standards for produce safety and for preventive controls during food processing that hold promise for reducing listeriosis.

Over time, many outbreaks have been linked to soft cheese made with unpasteurized milk, and FDA and Health Canada§§ estimate that the risk for listeriosis from soft-ripened cheeses is 50 to 160 times higher per serving when the cheese is made with unpasteurized milk rather than pasteurized milk. Nonetheless, investigations described in this report and elsewhere also have implicated cheeses made from pasteurized milk (9–11). Pasteurization eliminates *Listeria*, but contamination can occur after pasteurization. *Listeria* grows in moist environments, even at refrigeration temperatures, so it can thrive when soft cheeses that support its growth are contaminated. In addition to using pasteurized milk, soft cheese–making facilities need to use strict sanitation and microbiologic monitoring.

In the late 1990s and early 2000s, U.S. listeriosis incidence declined markedly after outbreak investigations prompted major industry and regulatory interventions, including using ingredients that inhibit growth of *Listeria* (12), to reduce contamination of processed meat (e.g., hot dogs and deli meat) (7). A risk assessment¶¶ presented for public comment by USDA-FSIS and FDA will inform efforts to reduce further *Listeria* contamination of ready-to-eat foods in retail settings.

FSMA calls on CDC to strengthen foodborne illness surveillance and outbreak response. States’ capacities vary considerably, and many lack sufficient staff and resources (13–15). CDC launched a collaborative network called FoodCore*** to develop methods to make outbreak detection and response faster, and the Integrated Food Safety Centers of Excellence,††† to provide technical assistance and training of public health staff in other states. As more states use the *Listeria* Initiative to gather data on cases quickly, outbreak response improves. Faster investigations save lives.§§§ During a 2011 multistate outbreak, the *Listeria* Initiative led to identification of cantaloupe as the food vehicle, and halt of its distribution, in less than 2 weeks (8); the response was much faster than previous investigations of large outbreaks of listeriosis, such as a 1985 outbreak associated with Mexican-style cheese in which 31 days elapsed between outbreak detection and product recall (16). Advanced laboratory methods will modernize diagnostics and surveillance; more outbreaks might be detected faster using real-time whole genome sequencing (17).

Consumers at higher risk for listeriosis and those who prepare their food can reduce their risk. Basic food safety measures (e.g., Clean, Separate, Cook and Chill¶¶¶) reduce the risk for listeriosis and other potentially serious infections. Persons at higher risk should follow the guidance for the general population not to consume unpasteurized milk or dairy products made from unpasteurized milk (e.g., soft cheese). They also should be aware that some Mexican-style soft cheeses made from pasteurized milk, like queso fresco, have been identified as a source of listeriosis. In addition, health-care providers are uniquely positioned to provide credible information about listeriosis prevention to patients at higher risk. Detailed advice on safely selecting, preparing, and refrigerating foods prone to *Listeria* contamination and other pathogens is available in English and Spanish at http://www.cdc.gov/*listeria*, http://www.fsis.usda.gov/fact_sheets/listeria_monocytogenes/index.asp, and http://www.fda.gov/food/resourcesforyou/consumers/ucm079667.htm.

**Key Points**

- *Listeria monocytogenes* infection (listeriosis) is a rare foodborne disease that often leads to bacteremia, meningitis, hospitalization, fetal loss, and death.
- Careful attention to food safety is especially important for older adults, pregnant women, and persons with immunocompromising conditions because almost all cases of listeriosis occur among these three groups at higher risk.
- The average annual incidence of listeriosis for the period 2009–2011 (0.29 cases per 100,000 population) indicates that no progress in reducing the rate of listeriosis has occurred in over a decade.
- Foods associated with recent listeriosis outbreaks, especially soft cheese and raw produce, highlight food safety gaps that can be addressed by industry, regulatory authorities, food preparers, and consumers.
- Additional information is available at http://www.cdc.gov/vitalsigns.

## Figures and Tables

**TABLE 1:** Demographic and clinical characteristics of cases of invasive *Listeria* infection (listeriosis), by risk group — *Listeria* Initiative, United States, 2009–2011

			Not pregnancy-associated					
			
	Pregnancy-associated*		Patients aged <65 yrs		Patients aged ≥65 yrs		Total	
				
Characteristic	No.	(%)†	No.	(%)†	No.	(%)†	No.	(%)†
**Total**	**227**	**(100)**	**474**	**(100)**	**950**	**(100)**	**1,651**	**(100)**
Female sex	227	(100)	218	(46)	489	(51)	**910**	**(55)**
Hispanic ethnicity§	85	(43)	77	(20)	54	(7)	**216**	**(16)**
Isolate source¶								
Blood	150	(66)**	334	(70)	824	(87)	**1,308**	**(79)**
CSF	41	(18)**	119	(25)	98	(10)	**258**	**(16)**
Other sterile site††	NA	—	23	(5)	35	(4)	**58**	**(4)**
Product of conception††	36	(16)	NA	—	NA	—	**36**	**(2)**
Hospitalization§§	133	(90)	417	(93)	850	(94)	**1,400**	**(93)**
Death or fetal loss¶¶	46	(21)***	53	(14)	193	(24)	**292**	**(21)**

**Abbreviations:** CSF = cerebrospinal fluid; NA = not applicable.

* Pregnancy-associated cases include those in pregnant women, fetuses, and infants aged ≤31 days.

† Percentages may not sum to 100 because of rounding.

§ Among 1,327 (80%) patients with available ethnicity data.

¶ When *L. monocytogenes* is isolated from multiple anatomical sites, a single site is reported (priority order: CSF, blood, other normally sterile site, and products of conception).

** Isolates from neonatal blood (n = 72), maternal blood (69), and both (nine); isolates from neonatal CSF (38), maternal CSF (two), and both (one).

†† For non–pregnancy-associated cases, other sterile sites were pleural fluid (n = 18 isolates), peritoneal or ascites fluid (14), joint or synovial fluid (nine), brain tissue (three), aortic tissue (one), eye (one), liver abscess (one), lung tissue (one), and pericardial fluid (one). For pregnancy-associated cases, products of conception were placental tissue (31) and amniotic fluid (five).

§§ Hospitalizations among singleton neonates for 147 pregnancy-associated cases and among 1,358 non–pregnancy-associated cases with data available.

¶¶ Deaths or fetal losses among singleton neonates for 224 pregnancy-associated cases and among 1,179 non–pregnancy-associated cases with data available.

*** Forty fetal losses and six neonatal deaths.

**TABLE 2:** Reported outbreaks of *Listeria* infection (listeriosis) — Foodborne Disease Outbreak Surveillance System, United States, 2009–2011

Year	Multistate	Total cases*	Consumption setting	Implicated food vehicle
**2009**	Yes	18	Private homes	Mexican-style cheese†
	Yes	8	Private homes§	Mexican-style cheese†
**2010**	No	8	Private homes	Hog head cheese¶
	No	2	Private homes	Sushi rolls (unspecified)
	No	4	Hospital food service	Undetermined
	No	10	Hospital food service	Pre-cut celery
	Yes	6	Private homes§	Mexican-style cheese†
**2011**	No	2	Unknown	Undetermined
	No	2	Private home and restaurant	Chive cheese† and ackawi cheese†**
	Yes	147	Private homes	Whole cantaloupe
	No	2	Private homes	Mexican-style cheese†
	Yes	15††	Wedding banquets	Aged, blue-veined cheese§§

* Total cases include laboratory-confirmed and epidemiologically linked cases.

† Soft cheese made from pasteurized milk.

§ *L. monocytogenes* isolates from these two outbreaks were indistinguishable by pulsed-field gel electrophoresis. The Food and Drug Administration sought a permanent injunction against the manufacturer after the first outbreak. The owners moved the manufacturing facility to a nearby location and reopened under a new name.

¶ Hog head cheese is a meat jelly made from swine heads and feet (i.e., it is not a dairy product).

** Ackawi is a white brine cheese.

†† Fourteen cases of febrile gastroenteritis (noninvasive, not culture-confirmed) and one case of culture-confirmed invasive disease reported.

§§ A blue cheese that was made from unpasteurized milk and aged for 60 days.

## Footnotes

* The main purpose of the *Listeria* Initiative is to facilitate outbreak investigations. Food exposure frequencies from cases associated with suspected outbreaks (identified by PulseNet) are compared with food history data from sporadic cases. This facilitates rapid identification and recall of contaminated foods. Additional information on the *Listeria* Initiative is available at http://www.cdc.gov/listeria/pdf/listeriainitiativeoverview_508.pdf.

† PulseNet, the national molecular subtyping network, subtypes all *L. monocytogenes* isolates using pulsed-field gel electrophoresis (PFGE). PulseNet is a network of laboratories in local, state, and federal health and regulatory agencies that use standard protocols, equipment, and nomenclature to test bacteria and submit their PFGE pattern combinations to a central database for comparison with one another. For *L. monocytogenes*, two patterns are submitted for most isolates (one for each of two restriction enzymes, *Asc*I and *Apa*I). Additional information about PulseNet is available at http://www.cdc.gov/pulsenet. Data on the proportion of patients with isolates submitted to PulseNet whose illnesses are reported to the *Listeria* Initiative are available at http://www.cdc.gov/listeria/pdf/listeria-annual-summary-2011-508c.pdf.

§ FoodNet is part of CDC’s Emerging Infectious Diseases Program (http://www.cdc.gov/foodnet).

¶ Additional information on national outbreak reporting is available at http://www.cdc.gov/nors.

** Listeriosis outbreaks were included in this report based on the date of onset of the first illness; an outbreak associated with raw sprouts, which began in 2008 and extended into 2009, was not included.

†† Additional information about FSMA, proposed standards for produce safety, and preventive controls during food processing is available at http://www.fda.gov/food/guidanceregulation/fsma/default.htm.

§§ Information about the *Quantitative Assessment of the Risk of Listeriosis From Soft-Ripened Cheese Consumption in the United States and Canada* available at http://www.fda.gov/downloads/food/foodscienceresearch/ucm338617.pdf.

¶¶ Additional information is available at http://www.fsis.usda.gov/science/risk_assessments/index.asp.

*** Additional information available at http://www.cdc.gov/foodcore/about.html.

††† Additional information available at http://www.cdc.gov/foodsafety/fsma.html.

§§§ An estimated 36 illnesses, 35 hospitalizations, and seven deaths were averted by recall of contaminated cantaloupe and consumer warnings (CDC, unpublished data, 2013).

¶¶¶ Additional information about the basics of food safety available at http://www.foodsafety.gov/keep/basics.
